# S53P4 BIOACTIVE GLASS PUTTY IN THE LOCAL TREATMENT OF CAVITARY CHRONIC OSTEOMYELITIS

**DOI:** 10.1590/1413-785220233101e258453

**Published:** 2023-02-20

**Authors:** GABRIELA NAGY BALDY DOS REIS, GABRIEL TROVA CUBA, WALTER HAMILTON DE CASTRO TARGA, PAULO SÉRGIO CONTADOR MIRAS, JOSÉ CARLOS BONGIOVANNI, MAURO JOSÉ SALLES, FERNANDO BALDY DOS REIS, ADRIANA MACEDO DELL’AQUILA

**Affiliations:** 1 Universidade Federal de São Paulo, Department of Orthopedics and Traumatology, São Paulo, SP, Brazil.; 2 Universidade Federal de São Paulo, Paulista School of Medicine, São Paulo, SP, Brazil.; 3 Universidade de São Paulo, School of Medicine, Institute of Orthopedics and Traumatology, São Paulo, SP, Brazil.; 4 Hospital Municipal Dr. Mário Gatti, Campinas, SP, Brazil.; 5 Universidade de Mogi das Cruzes, Department of Orthopedics and Traumatology, Mogi das Cruzes, SP, Brazil.

**Keywords:** Bioactive Glass S53P4, Biocompatible Materials, Bone Substitute, Chronic Osteomyelitis, Staphylococcus Aureus, Vidro Bioativo S53P4, Materiais Biocompatíveis, Substitutos Ósseos, Osteomielite Crônica, Staphylococcus Aureus

## Abstract

**Objective:**

Evaluating the clinical results of bioactive glass S53P4 putty for the treatment of cavitary chronic osteomyelitis.

**Methods:**

Retrospective observational study, including patients of any age with clinical and radiological diagnosis of chronic osteomyelitis, who underwent surgical debridement and implantation of bioactive glass S53P4 putty (BonAlive^®^ Putty, Turku, Finland). Patients who underwent any plastic surgery on the soft tissues of the affected site or had segmental bone lesions or septic arthritis were excluded. Statistical analysis was performed using Excel^®^. Demographic data, as well as data on the lesion, treatment, and follow-up, were collected. Outcomes were classified as “disease-free survival,” “failure,” or “indefinite.”

**Results:**

This study included 31 patients, of which 71% were men and had with a mean age of 53.6 years (SD ± 24.2). In total, 84% were followed-up for at least 12 months and 67.7% had comorbidities. We prescribed combination antibiotic therapy for 64.5% of patients. In 47.1%, *Staphylococcus aureus* was isolated. Finally, we classified 90.3% of cases as “disease-free survival” and 9.7% as “indefinite.”

**Conclusion:**

Bioactive glass S53P4 putty is safe and effective to treat cavitary chronic osteomyelitis, including infections by resistant pathogens, such as methicillin-resistant *S. aureus*. ***Level of Evidence IV, Case Series.***

## RESUMO

**Objetivo:**

Avaliar a atividade do vidro bioativo S53P4 em pasta no tratamento de osteomielite crônica.

**Métodos:**

Estudo observacional retrospectivo, com inclusão de indivíduos de qualquer idade com diagnóstico clínico e radiológico de osteomielite que realizaram tratamento cirúrgico com limpeza e desbridamento, seguido do preenchimento da cavidade com biovidro S53P4 em pasta (BonAlive^*®*^ Putty, Turku, Finland). Foram excluídos pacientes submetidos a procedimentos de cirurgia plástica nos tecidos moles do local afetado, com lesões ósseas segmentares e com presença de artrite séptica. A análise estatística foi realizada em Excel^*®*^ . Foram coletados dados demográficos, sobre a lesão, o tratamento e o acompanhamento. O desfecho foi classificado em “sobrevida livre de doença”, “falha” ou “indeterminado”.

**Resultados:**

Dos 31 pacientes analisados, 71% eram homens, com idade média de 53,6 anos (DP ± 24,26). Do total, 84% foram acompanhados por no mínimo 12 meses, e 67,7% apresentaram comorbidades. A terapia antibiótica combinada foi realizada em 64,5% dos pacientes, sendo o patógeno mais frequente o Staphylococcus aureus (47,1%). Ao final, 90,3% dos pacientes obtiveram “sobrevida livre de doenças” e 9,7% foram considerados “indeterminados”.

**Conclusão:**

O vidro bioativo S53P4 em pasta é seguro e eficaz no tratamento da osteomielite cavitária e de infecções por patógenos resistentes, incluindo o S. aureus multirresistente. **Nível de Evidência IV, Série de Casos.**

## INTRODUCTION

Among all types of osteomyelitis, the chronic form has a higher risk of recurrence. Chronic osteomyelitis occurs due to the intracellular invasion of microorganisms in osteoclasts, osteoblasts, and osteocytes and causes biofilm formation, persistent bone sequestration, and continuous bone resorption.^1^ Bone sequestration can create an infectious niche, in which bacteria perpetuate in biofilms, hindering the immune response and the action of systemic antibiotics. Therefore, a successful treatment depends on the resection of the bone sequestration and the consequent eradication of the microorganism involved.^1^

Surgical debridement removes the dead bone and biofilm, but produces bone defect. Bone lesions may have cavitary and segmental formation. Bone substitutes usually fill the bone defect.^2^ Besides providing structural strength, the ideal substitute must have three attributes to enable bone recovery: (1) osteoconduction, (2) osteoinduction, and (3) osteogenesis.^2^ Osteoconduction provides a biocompatible structure that works as a structural matrix for the adhesion of osteogenic cells and the growth of new blood vessels.^2^ Osteoinduction supports mitogenesis of undifferentiated mesenchymal cells, forming osteoprogenitor cells able to form new bone.^2^ Osteogenesis occurs when the graft material has cells capable of synthesizing a new bone. This property can only exist in the autograft or when bone substitutes are enriched with cultured autologous cells.^2^ ^),(^ ^3^ A new generation of biomaterials, called “bioactives,” emerged with better biological interaction with bone tissue and bioactive glass is among them.^4^ This bioglass works as a bone substitute and has shown *in vitro* the ability to inhibit bacterial growth without the use of antibiotic substances.^5^

Bioactive glass S53P4 (BonAlive^®^ Putty, Turku, Finland) consists of natural elements, as its composition includes 53% silicon dioxide (SiO_2_), 23% sodium oxide (Na_2_O), 20% calcium oxide (CaO), and 4% phosphorus pentoxide (P_2_O_5_).^6^ This biomaterial promotes osteoinduction and osteoconduction and attaches firmly to the living tissue, facilitating the growth of bone tissue, due to a chemical bond with the surrounding bone, and enabling the formation of a new bone.^6^ Moreover, it inhibits the growth of several species of plankton and biofilm-forming bacteria without the need for local antibiotic compounds. Studies show that its antibacterial properties result from increased local pH levels and, consequently, increased osmotic pressure, due to the exchange of alkaline ions with protons in solution in body fluid.^7^

The bioglass forms a chemical bond with the bone, but can also bond with soft tissues.^8^ Active bioglasses can come in the form of granules or putty. Considering their property of osteoinduction, heterotopic ossification must be avoided during its use.^8^ The formation of fistulas similar to those caused by chronic osteomyelitis is a possible manifestation.^9^ Bioactive glass putty could facilitate the filling of the bone defect, providing lower risk of the product to bond with soft tissues. This study aimed to evaluate the clinical use of bioactive glass S53P4 putty (BonAlive^®^ Putty, Turku, Finland) for the treatment of cavitary bone defects in patients diagnosed with chronic osteomyelitis.

## MATERIALS AND METHODS

### Study design and population

This retrospective observational cohort study was performed in a private tertiary care hospital in the municipality of São Paulo, São Paulo, Brazil. All participants signed an informed consent form. This study was approved by the Research Ethics Committee of the coordinator hospital under CAAE 77277617.0.1001.5455 on 02/19/2018.

All patients who used bioactive glass S53P4 putty (BonAlive^®^ Putty, Turku, Finland) for the treatment of osteomyelitis were identified by the orthopedic team. The inclusion criteria were: (1) patients of any age; (2) clinical (fistulas and pus at the site of the original bone lesion and dehiscence of the surgical wound) and radiological diagnosis (soft tissue edema, bone demineralization, periosteal reaction, and/or trabecular and cortical osteolysis) of chronic osteomyelitis; (3) having undergone surgery for debridement of the affected tissue and filling of the resulting cavity or segment with bioactive glass S53P4 putty from April 2017 to November 2019. The exclusion criteria were: (1) having undergone plastic surgery on the soft tissues of the site affected by osteomyelitis; (2) patients with segmental bone lesions (measuring < 2 cm, 2-5 cm, or > 5 cm); (3) having septic arthritis associated with osteomyelitis.

### Clinical data collection

Patient data were collected by the review of medical records. Clinical information included demographic characteristics, infected bones, comorbidities of patients and their life habits, antimicrobials relevant for prophylaxis and empirical and specific therapies, microbiological results of sample collections performed intraoperatively, duration of treatment, and follow-up time. Among comorbidities, diabetes, heart disease, neoplasia, paraplegia, tetraplegia, and thrombosis were analyzed. Clinical follow-up was performed by the orthopedic and trauma team that performed the surgery. Data collected during outpatient visits were used to classify the outcome of patients as “disease-free survival,” “failure,” or “indefinite.”

### Definitions

Criteria for defining osteomyelitis are not uniform in the scientific literature. In this study, the following criteria were used: (1) acute osteomyelitis as a surgical site infection detected within 30 days after trauma and chronic bone infection diagnosed after this period; (2) outcome classified as “disease-free survival” when the patient recovered without signs or symptoms of osteoarticular infection and the need for antibiotics or surgery to treat bone infection; outcome classified as “indefinite” in the case of loss of bone segment, death, or amputation due to vascular insufficiency; outcome classified as “failure” in the case of need for additional antimicrobial surgery or therapy; (3) considering only the collection of soft tissue and bone samples; (4) polymicrobial bone infection defined as the isolation of two or more microorganisms in at least one soft tissue or bone tissue sample or monomicrobial infection described as the identification of only one pathogen in these culture samples; (5) bacterial multiresistance, such as resistance of microorganisms to at least two classes of antibiotics, and detected in the hospital by the standardized sensitivity test.

### Microbiological criteria

Soft tissue and/or bone samples were collected after extensive surgical debridement of the infectious focus, inserted in identified sterile jars, and then sent to the microbiology laboratory of the hospital, where they were cultured and identified using traditional microbiological techniques.

### Statistical analysis

In statistical analysis, all data were initially entered in an Excel table. Categorical data were presented as absolute and percentage numbers and the continuous variables were presented as median.

## RESULTS

We analyzed 31 patients, of which 71% were men and had with a mean age of 53.6 years (SD ± 24.26 years). Most patients (84%) were followed up for at least 12 months, with a minimum period of six months, maximum of 39 months, and average of 22 months (SD ± 8.81 months).

In 93.5% of cases, lower limbs were affected, including fractured ankle (32.2%), foot bones (16.1%), femur (12.9%), fibula (12.9%), humerus (6.5%), tibia (6.5%), acetabulum (6.5%), and hip (6.5%). A total of 9.7% of patients had pseudoarthrosis and 19.4% had fistulas. All patients had chronic osteomyelitis: 48.4% had infection with *in situ* osteosynthesis and 51.6% infection without synthesis material. The infection occurred up to three months after surgery in 58% of patients and after more than three months in 42%.

Table 1 shows the comorbidities observed. In total, 67.7% of patients had one or more comorbidities. Hypertension (38.7%) and diabetes (32.3%), followed by neoplasia (6.5%), were the most prevalent comorbidities. No patient was a smoker or alcoholic or used immunosuppressive drugs.

**Table 1:** Distribution of patient comorbidities.

Comorbidity	n	%
Systemic arterial hypertension	12	38.7
Diabetes mellitus	10	32.3
Neoplasia	2	6.5
Paraplegia	1	3.2
Tetraplegia	1	3.2
Thrombosis	1	3.2

Regarding the proposed treatment, Table 2 shows that most patients (64.5%) underwent combination systemic antibiotic therapy. Teicoplanin and meropenem (30%) was the most used combination, followed by clindamycin and ceftriaxone (25%). The maximum duration of systemic antibiotic therapy was six weeks and teicoplanin was the most used antibiotic (44.8%). Two patients (6.5%) did not undergo systemic antibiotic therapy.

**Table 2:** Use of antibiotic therapy after surgical cleaning.

Antibiotic therapy	n	%
Did not undergo	2	6.5
Monotherapy	9	29.0
Combination therapy	20	64.5
**Antibiotics used**	**n**	**%**
Teicoplanin	13	41.9
Meropenem	9	29.0
Daptomycin	7	22.6
Ceftriaxone	6	19.4
Clindamycin	5	16.1
Other	11	35.5

**Figure 1:**
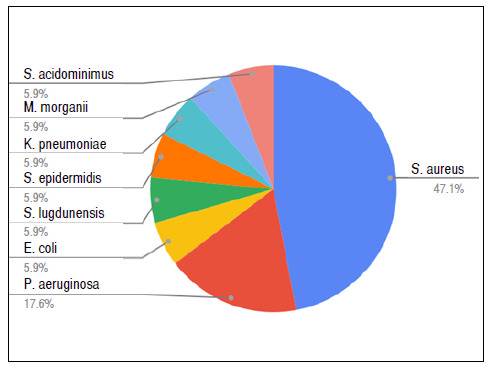
Infectious agents identified by soft tissue and bone tissue cultures collected during surgeries.

We collected deep soft tissue and bone fragment samples of all patients for culture analysis and 51.6% were positive. Two patients had polymicrobial infection (two pathogens identified). Figure 1 shows that *Staphylococcus aureus* (47.1%) was the most frequent agent, followed by *Pseudomonas aeruginosa* (17.6%).

**Figure 2:**
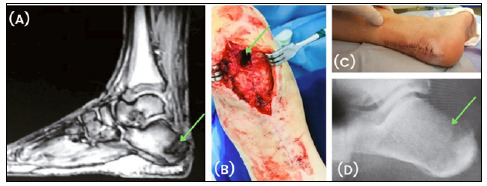
Calcaneus with osteomyelitis treated with bioactive glass S53P4 as a bone substitute: (A) preoperative magnetic resonance image showing osteomyelitis in the calcaneus (arrow); (B) intraoperative image showing the lesion (arrow); (C) image three weeks after surgery; (D) radiography showing bioactive glass S53P4 in the treated bone cavity (arrow) five months after surgery.

Regarding the prospective follow-up time, we followed up 83.9% of patients (n = 26) for more than one year and 48.4% (n = 15) for at least two years. We followed up only 16.1% of patients (n = 5) from six to 11 months. For 90.3% (n = 28), the primary outcome of the study was “disease-free survival.” We followed up 85.7% of those (n = 24) for at least one year. The outcome of only 9.7% of patients (n = 3) was “indefinite.” Of these, one case resulted in amputation due to vascular insufficiency and the other two evolved to death unrelated to bone infection (neoplasia). No patient presented heterotopic ossification. Figure 2 shows the treatment of a patient with cavitary chronic osteomyelitis in the calcaneus treated with surgical implantation of bioactive glass S53P4. During outpatient follow-up, images showed cavitary filling in the calcaneus three weeks and 20 weeks after surgery. These controls and the clinical picture did not present signs of recurrence of the infection.

## DISCUSSION

This study showed the possibility of treating osteomyelitis with bioactive glass S53P4 putty. In this study, in association with systemic antibiotic therapy, which was used for a relatively short time, bioactive glass S53P4 putty was effective for the treatment of osteomyelitis in 90.3% of patients and no patient presented heterotopic ossification. This finding is similar to other studies on the use of bioglass granules, which showed success rates in the treatment of osteomyelitis in 90% of cases.^7^ ^),(^ ^10^ ^)-(^ ^12^

In the conventional treatment of patients with osteomyelitis, in which bone substitutes with orthopedic cement (polymethylmethacrylate) and local antibiotics have similar high success rates, multiple extra surgeries are necessary for the removal of the polymer.^7^ The possible necrosis of bone tissue due to exothermic injury and fat embolism are other disadvantages of the use of polymers.^3^ In the treatment with bioglass, only one surgical procedure is sufficient. Therefore, the chance of comorbidities is lower, health costs are lower, and the length of hospital stay is short.^13^ Moreover, bioactive glass S53P4 allows the remodeling of the natural bone over time, which ensures the conservation of bone stock.^11^ This is important because many patients with chronic osteomyelitis may need additional surgeries throughout life.

Multiple surgical procedures and diabetes influence the risk of infection in orthopedic surgery^13^ and the infection rate in the presence of implants is usually higher.^14^ In this study, one third of patients had diabetes and half of them had synthesis material, and the bioglass used was able to treat bone infection.

Previous studies show that the bond between bioglass and bone forms more rapidly when the bioactive glass has 45-52% SiO_2_ by weight. This glass form a chemical bond with the bone, but also with soft tissues.^8^ Bioglasses with 55-60% SiO_2_ react more slowly, last more, have bioactivity, and do not bond with soft tissues. Depending on the composition of the bioglass, especially its percentage of SiO_2_, its bond with soft tissues may favor heterotopic ossification.^8^

Bioglass granules or putty present antimicrobial activity against gram-positive and gram-negative bacteria and do not select resistance to microbial strains,^15^ which makes them ideal bone substitutes for the treatment of bone infections, including in the presence of multiresistant strains.^15^ *In vitro* bioglass acts against diverse agents, even in osteomyelitis and infections related to prostheses caused by multiresistant organisms; thus, bioglass is antibacterial.^5^ In this study, we evaluated the clinical evolution of patients treated with bioglass putty in association with systemic antibiotics and observed the antimicrobial action of bioactive glass S53P4 and a favorable evolution in bone infections caused by *S. aureus*, *P. aeruginosa*, *Escherichia coli*, *Staphylococcus lugdunensis*, *Staphylococcus epidermidis*, *Klebsiella pneumoniae*, *Streptococcus acidominimus*, and *Morganella morganii*.

In line with previous studies, *S. aureus* was the most common agent (47.1%) in bone infections.^16^ The use of bioglass putty was safe, as its antimicrobial activity makes it capable of eradicating oxacillin-sensitive and -resistant *S. aureus* infections.

For many years, the treatment of bone infections was based on prolonged use of antimicrobials.^17^ Patients usually underwent long antibiotic therapies, which could last up to six months for staphylococcal infections.^18^ However, several studies show that shorter treatments may be appropriate for most cases of prosthetic joint infection or osteomyelitis^19^ and may be associated with a reduction in the length of hospital stay, incidence of adverse events, and predisposition to proliferation of multiresistant microorganisms.^20^ Several clinical trials evaluated 4-, 6-, or 12-week therapies,^19^ ^),(^ ^20^ aiming to reduce the time of antibiotic use. In this study, we used bioglass putty as an adjuvant in the treatment of bone infections with and without implants. The maximum antibiotic therapy time observed in this study was six weeks and two patients did not underwent this treatment.

As this was a retrospective study, in which we extracted data from medical records, we could not diagnose bones anatomopathologically. We based the diagnostic criterion for osteomyelitis on clinical, microbiological, and radiological criteria.

## CONCLUSION

Bioactive glass S53P4 putty was safe and effective for the treatment of osteomyelitis and no patient presented heterotopic ossification. This bioactive glass was capable of eradicating infection caused by several types of bacteria, including multiresistant *S. aureus*, which is the main agent in osteoarticular infections.
